# Progressive vitiligo induced by recurrent melanoma

**DOI:** 10.1002/ccr3.5290

**Published:** 2022-01-17

**Authors:** Yasuo Nakai, Ayaka Ueki, Keiichi Yamanaka

**Affiliations:** ^1^ Department of Dermatology Mie University Graduate School of medicine Tsu Japan

**Keywords:** local immunity, melanoma, recurrence, vitiligo

## Abstract

A woman had undergone excision for primary melanoma of the left heel and dissection of groin lymph nodes. The recurrent tumor on the lateral left lower leg developed six months ago and the depigmented plaques spread extensively on the left lower limb. The depigmented macules were localized to the left lower limb and were not seen in other areas. Although the left groin lymph node had been dissected, the local immune environment of anti‐tumor immunity was preserved. The cause of melanoma‐associated vitiligo is regarded to be anti‐tumor autoimmune mediated, and this phenomenon is recently recognized during the therapy with immune checkpoint inhibitors in the treatment of stage III and IV melanoma

## Graphical abstract

The recurrence of melanoma was detected on her left lower leg, and extensively developed depigmented plaques were localized on left lower limb. Although left groin lymph node was dissected, local environment of anti‐tumor immunity was preserved.

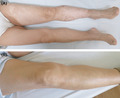

A 77‐year‐old woman consulted to our department because of the recurrence of melanoma on her left lower leg. She had undergone local excision for melanoma of the left heel and dissection of groin lymph nodes 10 years ago, followed by five courses of anticancer therapy and additional four years of immunotherapy with IFN‐β. The tumor on the lateral left lower leg developed six months ago and was histologically recurrence. However, the depigmented plaques that had appeared and developed at the same time had spread extensively on the left lower limb. The depigmented macules were localized with the left lower limb and were not seen in other areas (Figure 1A). Although the left groin lymph node had been dissected, the local immune environment of the lower limb would be preserved. During the following two months, the depigmentation appeared around the right knee (Figure 1B). The cause of melanoma‐associated vitiligo is regarded to be anti‐tumor autoimmune mediated,^1^ and the main effectors are cytotoxic CD8+ T cells that target melanocyte antigens responsible for melanin synthesis.^1^ This phenomenon is recently recognized during the therapy with immune checkpoint inhibitors in the treatment of stage III and IV melanoma.^2^

**FIGURE 1:**
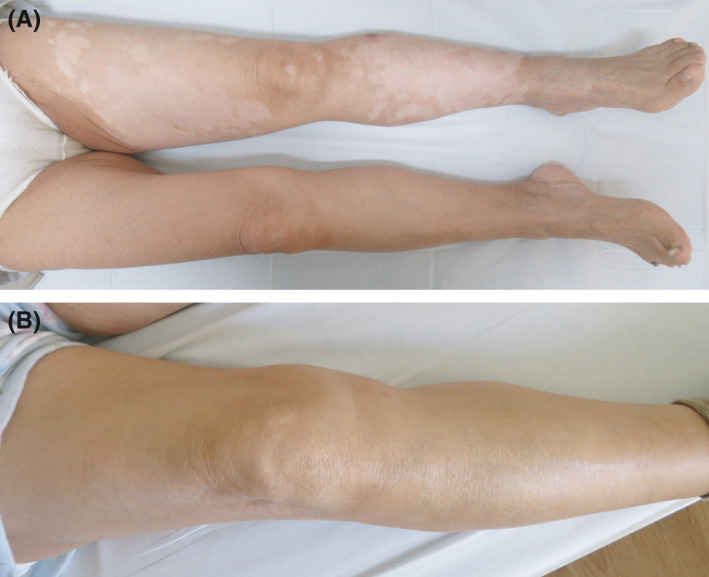
(A) The recurrent tumor was located on the left lower leg. The depigmented plaques had spread extensively within six months but limited to the left lower limb and were not seen in other areas. (B) During the following two months, the depigmentation appeared around the right knee

## ACKNOWLEDGEMENTS

None.

## CONFLICT OF INTERESTS

We declare no competing interests.

## AUTHOR CONTRIBUTIONS

NY, AU, and KY cared for the patient, wrote the manuscript. We all revised the manuscript.

## ETHICAL APPROVAL

The study was conducted in accordance with the Declaration of Helsinki. The patient provided written informed consent to publish the case, including the publication of images. The paper is exempt from ethics committee approval as only one case was reported.

## CONSENT

Written consent for publication was obtained from the patient.

## Data Availability

The patient's data is not publicly available on legal or ethical grounds.

## References

[1] Cui J , Bystryn JC . Melanoma and vitiligo are associated with antibody responses to similar antigens on pigment cells. Arch Dermatol. 1995;131:314‐318. 7887661

[2] Burzi L , Alessandrini AM , Quaglino P , Piraccini BM , Dika E , Ribero S . Cutaneous events associated with immunotherapy of melanoma: a review. J Clin Med. 2021;10(14):3047. doi:10.3390/jcm10143047 34300213 PMC8308045

